# First results of electrode reimplantation and its hypothetical dependence from artificial brain maturation

**DOI:** 10.1007/s00405-020-06125-1

**Published:** 2020-06-19

**Authors:** Willi Roßberg, Max Timm, Farnaz Matin, Alessandra Zanoni, Caroline Krüger, Alexandros Giourgas, Eva Bültmann, Thomas Lenarz, Andrej Kral, Anke Lesinski-Schiedat

**Affiliations:** 1 Otorhinolaryngology Department, Head and Neck Surgery, Hanover Medical University, Hannover, Germany; 2 Institute of Diagnostic and Interventional Neuroradiology, Hanover Medical University, Hannover, Germany; 3 Institute of AudioNeuroTechnology and Department of Experimental Otology, ENT Clinics, School of Medicine, Hanover Medical University, Hanover, Germany

**Keywords:** Cochlear implant, Reimplantation, Depth of insertion, Insertion angle

## Abstract

**Background:**

After introducing the first Cochlear Implants also in children theses are grown with electrical intracochlear stimulation and subsequent auditory cortical development. Over the meantime the positioning of the electrode was changed orientated on the development of electrode design, ability to insert atraumatic and on the widening of the indications towards highfrequency deafness.

**Methods:**

In this pilot study we analysed five prelingually deafened patients implanted as child in the late 90’s and had a reimplantation 2016 or later. We compared CT and DVT (cone beam CT) scans of the temporal bone and measured the insertion angle, the cochlear coverage, the total length of the electrode in the cochlea and the distance of the first active electrode to the round window. Moreover, we compared their speech understanding before and after reimplantation.

**Results:**

The results show a lowering in the insertion angle, the cochlear coverage, the total length of the electrode in the cochlea, in the distance of the first active electrode to the round window and in the speech understanding after reimplantation.

**Conclusion:**

These results show a difference in the depth of insertion while the speech understanding is not significantly improving in this group—although the technology is advanced. The influence of auditory maturation with CI in these patients will be discussed.

## Introduction

Cochlear implantation has become a routine procedure since the first patient with deafness was implanted in 1984. The implants restore the missing function of inner hair cells by transforming the acoustic signal into electrical stimuli for activation of auditory nerve fibres. Children can achieve a near to normal speech and language development when their deafness is detected early and implantation is performed thereafter.

Given the nature of electronic devices it is more than likely that some implants will fail over time. There were estimations that a cochlear implant has a life expectancy of more than 20 years. A congenitally deafened child implanted at a young age may need 3–4 different implants in their lifetime [1].

Indications for reimplantation are technical complications as hard or soft failures and medical complications. Technological upgrades are discussed as a new indication. Generally, reimplantation may be performed without major difficulties although potential medical complications are to damage the facial nerve, the sigmoid sinus, the internal carotid artery, the external wall of the auditory canal, the tympanic membrane, the dura or the ossicular chain. In general, it can be permitted by an adequate surgical technique. Another challenge are new tissue formations such as bone or connective around the electrode which complicate the extraction of the electrode and may require additional surgical procedures such as the use of a rigid probe electrode for bougienage of the cochlea, drill out techniques or use of split array electrodes [2]. If the new electrode is not completely inserted, the hearing results may be poorer than with the first implant [3].

Generally, an adequate cochlear coverage should be achieved with the electrode in order to stimulate the majority of spiral ganglia cells [2]. In the cochlea, high frequencies up to 20 kHz are represented in the base while low frequencies down to 20 Hz are located in the apex [4]. With an insertion depth of at least 360° a partial cochlear coverage can be achieved mainly for the high range and is achieved with most of the current electrode arrays. Some manufacturers postulate a higher cochlear coverage than 360° is needed to reach also the apical neuronal elements and stimulate lower frequencies [2].

In former decades cochlear implantation was still a rather new surgical technique. The insertion of the electrode was in most cases more traumatic, resulting in a loss of the residual hearing in many patients. Electrodes were inserted deeper far up into the second cochlear turn with the estimation of a sufficient electrical stimulation of neurons leaving out the most basal part of the cochlea [5]. Current electrode arrays are designed to reduce the insertion trauma and improve the location in the cochlea either at the lateral wall or in a perimodiolar position. The surgical technique was also further developed to protect the intracochlear structures and preserve the residual hearing [6].

Given the advances both in technology and cochlear implant surgery the question arises weather and how early implanted congenitally deaf patients might benefit from (upgrade) reimplantation.

This study focuses on the insertion depth of cochlear implant electrodes before and after reimplantation and the effect of electrode location on speech understanding.

## Materials and methods

For this pilot study we evaluated five prelingually deafened children, three of them male and two of them female, age 2.1–6.9 years who had their first cochlear implantation in the late nineties and had a reimplantation 2016 or 2017. Four of the five patients are unilateral implanted, patient three received a second implant in the contralateral ear in 2008.

The reasons for reimplantation were technical problems as soft failure or hard failure, medical complications or a technological upgrade.

**Fig. 1:**
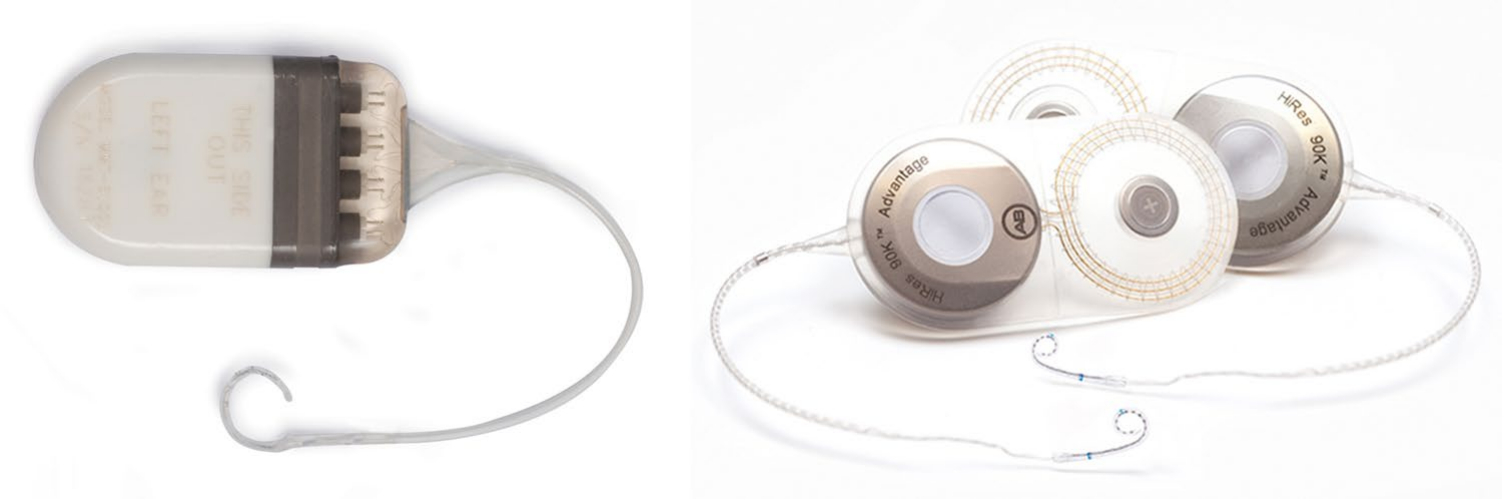
Comparison of an Advanced Bionics Clarion C1 implant and an Advanced Bionics HiRes90k Advantage (admission from Advanced Bionics)

The first implant was an AB Clarion C1 either implanted with or without a positioner, the second implant was an AB HiFocus MS electrode either with a HiRes 90K implant or an Ultra 3D implant (Fig. 1).

**Fig. 2:**
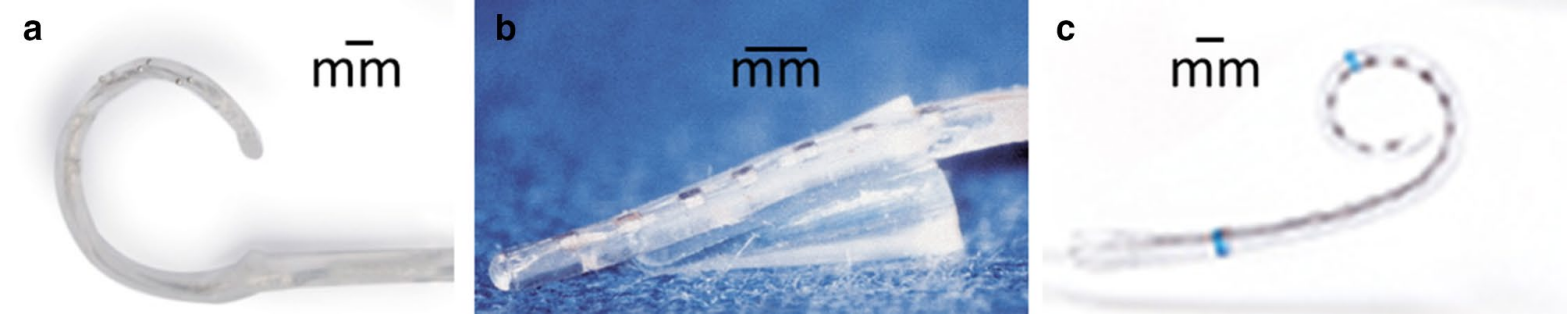
Different electrodes in use: **a** AB Clarion C1 electrode; **b** positioner; **c** AB Hi Focus MS electrode

In four of the five patients reimplantation was uneventful while in one patient the positioner had to be left in the cochlea due to new bone formation. The Hifocus MS electrode could be inserted completely without resistance in all five patients. The first electrode was straight and was inserted as deep as possible and in some cases further advanced moved towards the modiolus with the positioner. The second electrode by design was inserted only in the basal turn of the cochlea (Fig. 2).

**Fig. 3:**
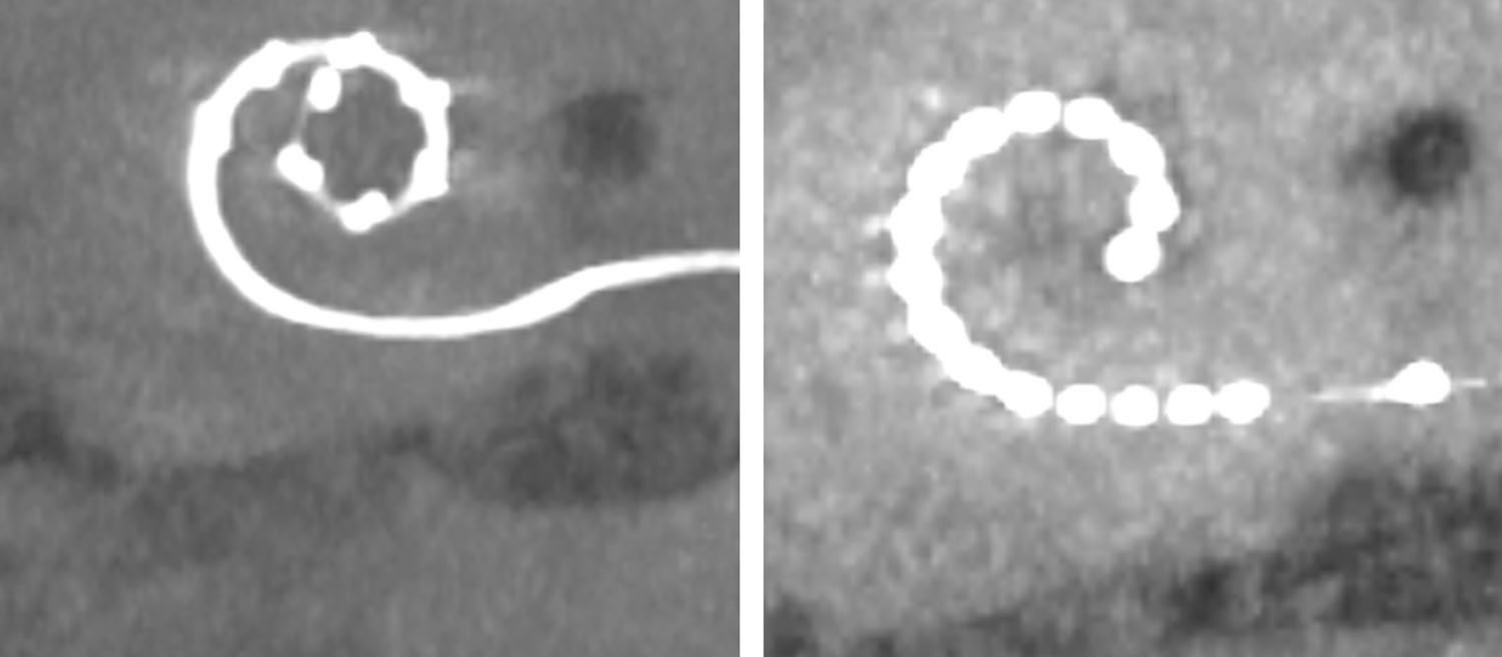
Imaging (Reconstruction of a full basal turn view of the left cochlea) of patient 4 before and after the reimplantation

**Fig. 4:**
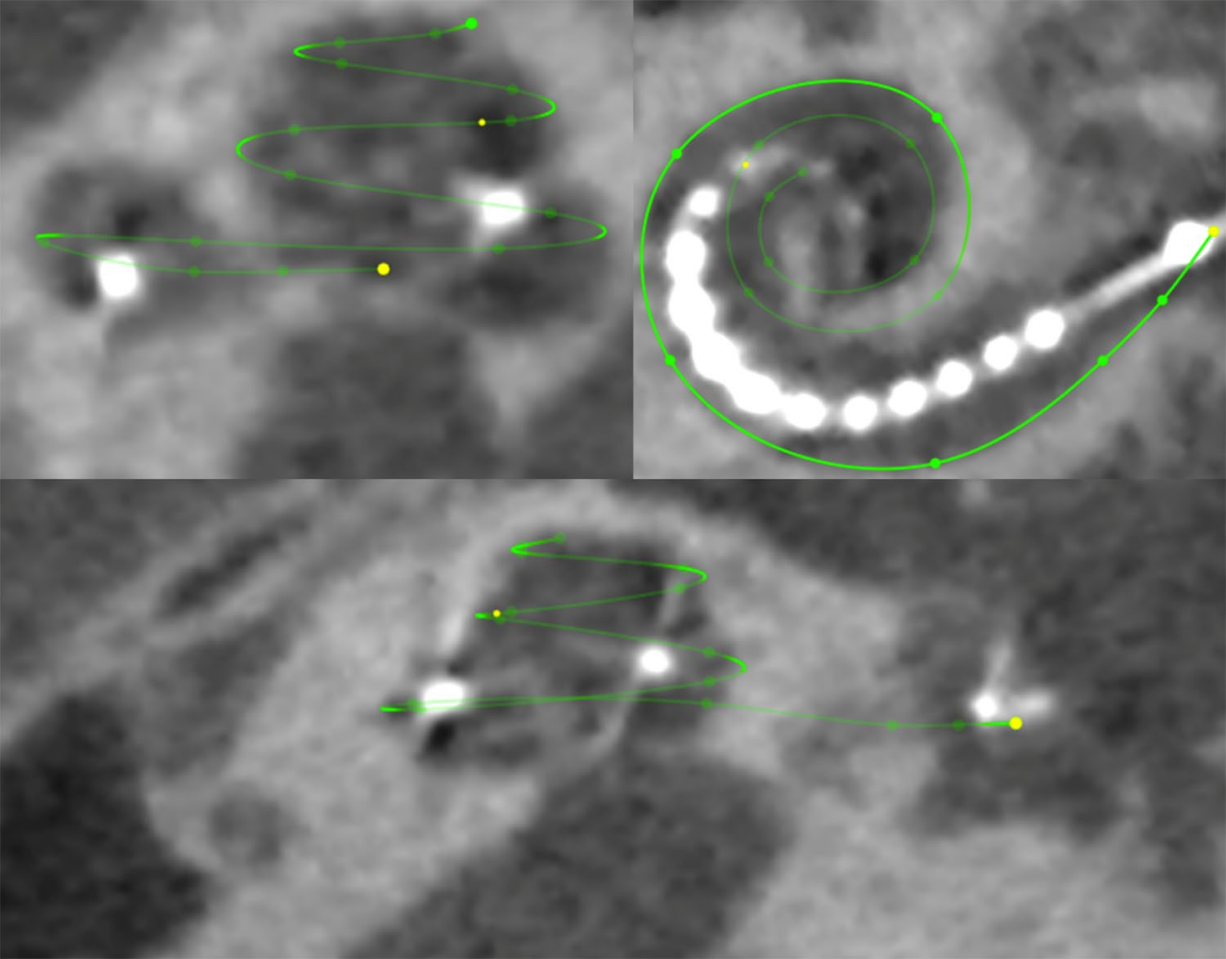
Image of the cochlea segmentation for the measurement of the CDL of patient 4

**Fig. 5:**
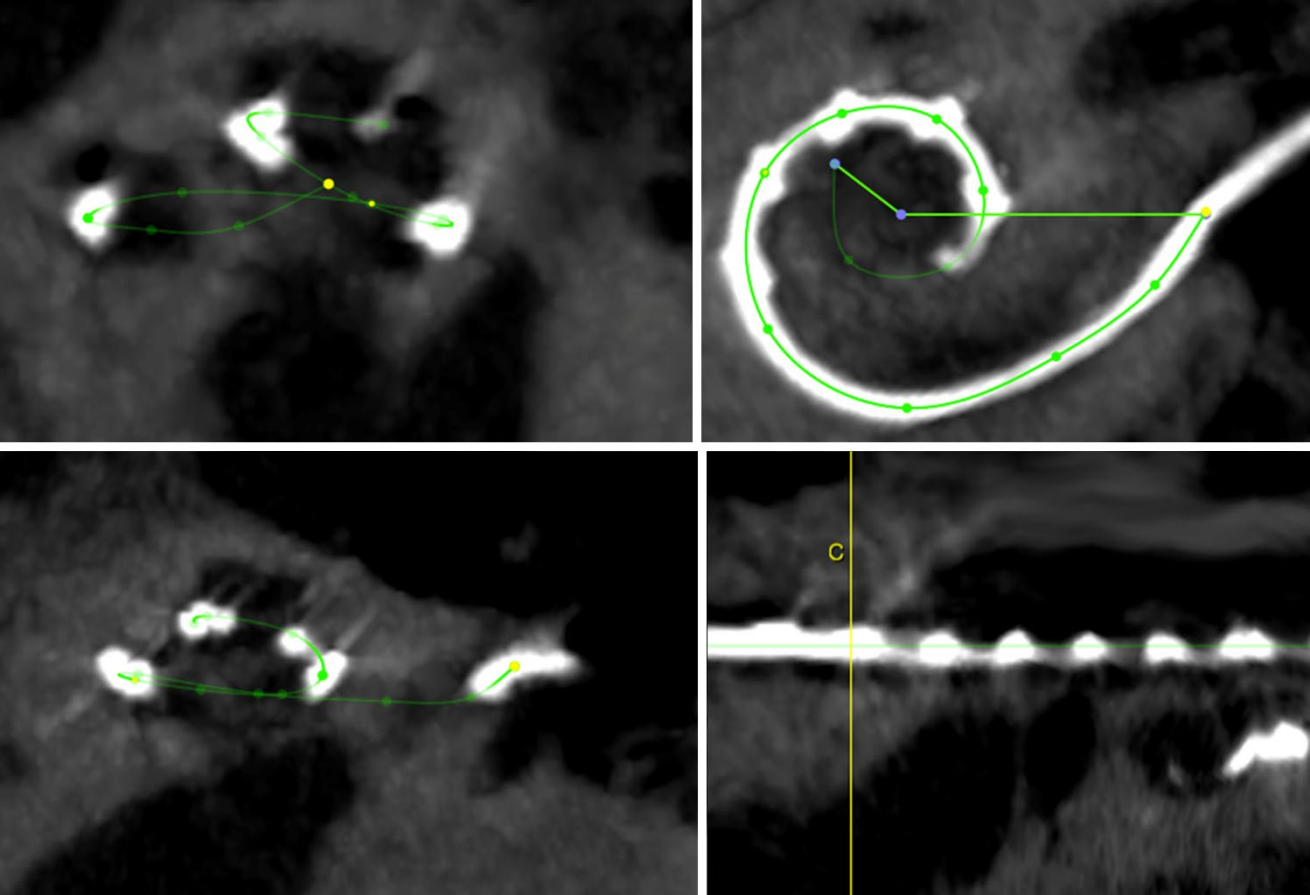
Image of the electrode array segmentation for the analysation of the localization of the first active contact and the CCL of patient 4

High resolution computer tomography (CT) or cone beam CT (digital volume tomography; DVT) scans of the temporal bone before and after the reimplantation were analysed with respect to differences in insertion angle, cochlear coverage and location of the first active electrode in the basal turn (Fig. 3). For these comparisons OsiriX MD software (version 2.5.1 64 bit, Pixmeo SARL, Switzerland) was used. The insertion angle (Ѳins) was measured by the line from the centre of the round window through the modiolar axis to the middle of the most apical electrode contact [7, 8]. Cochlear duct length (CDL), the cochlear coverage (CCL), the total length of the electrode in the cochlea and the measurement of the location of the first active electrode in the cochlear turn methods described by Schurzig et al. [8] were used by performing a cochlea and electrode array segmentation (Figs. 4, 5). The frequency range of the cochlea covered by the electrode array, i.e. the area along the cochlear partition between the most basal and apical contact of the electrode array was calculated using the frequency map for the human cochlear organ or corti as described by Stakhovskaya et al. [9].

For speech recognition we used the Freiburger monosyllabic with Cochlear Implant only before and one year after reimplantation.

Statistical analysis was done by using the Wilcoxon matched pairs test with SPSS (IBM SPSS Statistics 24) to see if these changings are significant.

Ethic approval is pronounced positively from MHH-Ethics committee.

## Results

*Patient 1 (female)* is prelingually deaf on both sides. She was implanted with an AB Clarion with positioner on the right side at the age of 3.3 years. With 20.1 years she got reimplanted with an AB HiRes 90k HiFokus MS electrode due to medical reasons. During the surgery the extraction of the positioner was not possible. The CDL is 36.5 mm. The angle of insertion (Ѳins) changed from 712° to 487° and the CCL from 0.92 to 0.71. The total length of the electrode in the cochlea changed from 25.1 to 17.9 mm while the distance of the first active electrode to the round window changed from 9.6 to 3.0 mm. The frequency map of the human cochlear organ of corti shows a frequency spectrum of 156 Hz to 3555 Hz for the old implant and 345 Hz to 12686 Hz for the new one. The speech reception dropped from 40% in the Freiburg monosyllabic test to 25% one year after reimplantation.

*Patient 2* (male) is prelingually deaf on both sides and was implanted with an AB Clarion without positioner on the right side at the age of 2.7 years. He got reimplanted at the age of 19.4 years with an AB HiRes 90k Adv. HiFokus MS due to a soft failure of the implant. The CDL of this patient is 38.7 mm. The Ѳins changed from 587° to 397° and the CCL from 0.78 to 0.61. The total length of the electrode in the cochlea changed from 23.2 to 17.7 mm while the distance of the first active electrode to the round window changed from 6.5 to 2.8 mm. The frequency map of the human cochlear organ of corti shows a frequency spectrum of 317 Hz to 10675 Hz for the old implant and 745 Hz to 11839 Hz for the new one. The speech reception changed from 30% in the Freiburg monosyllabic test to 35% one year after reimplantation.

*Patient 3* (female) is prelingually deaf on both sides. She was implanted with an AB Clarion with positioner on the right side at the age of 2.1 years. She got reimplanted at the age of 19.0 years with an AB HiRes Ultra HiFokus MS due to an upgrade. The CDL of this patient is 40.7 mm. The Ѳins changed from 576° to 374° and the CCL from 0.74 to 0.55. The total length of the electrode in the cochlea changed from 20.6 to 17.7 mm while the distance of the first active electrode to the round window changed from 4.4 to 3.0 mm. The frequency map of the human cochlear organ of corti shows a frequency spectrum of 110 Hz to 10197 Hz for the old implant and 821 Hz to 13592 Hz for the new one. The speech reception changed from 25% in the Freiburg monosyllabic test to 30% one year after reimplantation. Patient 3 got implanted on the contralateral side at the age of 10 years with an AB HiRes 90k Adv. HiFokus MS. She cannot understand monosyllabic words on that side.

*Patient 4* (male) is prelingually deaf on both sides and was implanted with an AB Clarion without positioner on the right side at the age of 3.5 years. He got reimplanted at the age of 24.3 years with an AB HiRes Ultra HiFokus MS due to a hard failure of the implant. The CDL of this patient is 41.7 mm. The Ѳins changed from 570° to 374° and the CCL from 0.74 to 0.58. The mean total length of the electrode in the cochlea changed from 25 to 17.7 mm while the distance of the first active electrode to the round window changed from 14.0 to 3.0 mm. The frequency map of the human cochlear organ of corti shows a frequency spectrum of 741 Hz to 3259 Hz for the old implant and 813 Hz to 11839 Hz for the new one. The speech reception changed from 35% in the Freiburg monosyllabic test to 30% one year after reimplantation.

*Patient 5* (male) is prelingually deaf on both sides. He was implanted with an AB Clarion without positioner on the right side at the age of 6.9 years. He got reimplanted at the age of 26.0 years with an AB HiRes 90K Adv. HiFokus MS due to a soft failure of the implant. The CDL of this patient is 37.3 mm. The Ѳins changed from 394° to 328° and the CCL from 0.63 to 0.56. The mean total length of the electrode in the cochlea changed from 20.0 to 17.1 mm while the distance of the first active electrode to the round window changed from 6.1 to 3.5 mm. The frequency map of the human cochlear organ of corti shows a frequency spectrum of 734 Hz to 7067 Hz for the old implant and 1097 Hz to 11704 Hz for the new one. The speech reception changed from 15% in the Freiburg monosyllabic test to 10% one year after reimplantation.

In total our data shows a lowering in the insertion angle, the cochlear coverage, the total length of the electrode in the cochlea and in the distance of the first active electrode to the round window and in the speech understanding after reimplantation in every patient. The speech understanding was in four of five patients about the same (± 5%) and one patient worsened clinical relevantly by 15%.

Table 1 shows the data of all five patients.

**Table 1:** Data of the five patients included into this study are shown

Patient	1		2		3	
Speech status	Prelingually Deaf		Prelingually Deaf		Prelingually Deaf	
Cause of hearing loss	Genetic		Unknown		Genetic	
Implanted side	Right		Right		Right	
CDL in mm	36.5		38.7		40.7	
	Old implant	New implant	Old implant	New implant	Old implant	New implant
Date implantation/reimplantation	8.6.1999	23.03.2016	12.1.2000	07.10.2016	16.3.2000	15.02.2017
Age implantation/reimplantation	3.3	20.1	2.7	19.4	2.1	19.0
Product	AB_Clarion with positioner	HiRes 90K HiFokus MS	AB_Clarion	HiRes 90K HiFokus MS	AB_Clarion with positioner	HiRes Ultra HiFokus MS
Ѳins in °	712	487	587	397	576	374
Insertation length in mm	25.1	17.9	23.2	17.7	20.6	17.7
CCL in mm	33.6	25.9	30.2	23.5	30.1	22.4
CCL in %	92.1	71.0	78.0	60.7	74.0	55.0
Distance 1st contact in mm	9.6	3.0	6.5	2.8	4.4	3.0
Lowest frequency in Hz	156	345	317	745	110	821
Highest Frequency in Hz	3555	12,686	10,675	11,839	10,197	13,592
Speech	40	25	30	35	25	30
Reason for reimplantation	Medical		Technical (soft failure)		Upgrade	
Other side	Unaided		Unaided		HiRes90k (2008)	
Complications reimplantation	Positioner stayed in cochlea		None		None	

Patient	4		5		Mean	
Speech status	Prelingually Deaf		Prelingually Deaf			
Cause of hearing loss	Unknown		Unknown			
Implanted side	Left		Right			
CDL in mm	41.7		37.3		39	
	Old implant	New implant	Old implant	New implant	Old implant	New implant
Date implantation/reimplantation	6.3.1996	07.12.2016	12.9.1997	26.09.2016		
Age implantation/reimplantation	3.5	24.3	6.9	26.0	3.7	21.8
Product	AB_Clarion	HiRes Ultra HiFokus MS	AB_Clarion	HiRes 90K HiFokus MS		
Ѳins in °	570	374	394	328	568	392
Insertation length in mm	25.0	17.7	20.0	17.1	23	18
CCL in mm	31.0	24.1	23.4	20.8	30	23
CCL in %	74.3	57.8	62.7	55.8	76	60
Distance 1st contact in mm	14.0	3.0	6.1	3.5	8.1	3.1
Lowest frequency in Hz	741	813	734	1097	412	764
Highest Frequency in Hz	3259	11,839	7067	11,704	6951	12,332
Speech	35	30	15	10	29	26
Reason for reimplantation	Technical (hard failure)		Technical (soft failure)			
Other side	Unaided		Unaided			
Complications reimplantation	None		None			

## Discussion

This pilot study focuses on reimplantation in early implanted congenitally deaf patients who undergo reimplantation with technologically far advanced implants. Electrodes at the first implantation in the late 90’s were deeply inserted with no electrical stimulation of the most basal part of the cochlea which can result in poor performance. The Second electrode was positioned mainly in the basal turn of the cochlea due to the changed electrode design over the years. This change in electrode position besides other technological advances might also have an impact on auditory performance. The missing stimulation of the most basal part of the cochlea during development of the auditory system with the first implant and the change of the electrode position with stimulation of so far not activated neural elements could lead to some undesired effects.

Reimplantation was first described by Hochmair-Desoyer and Burian [10]. Since that time, many studies were able to show that it is a safe surgical and fitting procedure and can be performed without complications in most cases [1, 2, 11–14]. New tissue and bone formation can make reimplantation difficult [2].

The extraction of the old electrode and reinsertion of the new is challenging. In cases, with new bone formations around the cochlear insertion is mechanically difficult. If electrodes with larger diameters shall be inserted, the complete insertion is sometimes difficult. If the electrode is not completely inserted, auditory performance may also be worse [2].

If the same implant and electrode is used and with the identical electrode position in the cochlea the hearing results after surgery are comparable. In cases of technological upgrade with reimplantation of a technically advanced implant and with improved speech processing and coding strategies, even better hearing results may be achieved [2, 15].

This is true for postlingually deaf patients. So far, there are no reports in literature on congenitally deaf patients being implanted in childhood and reimplanted as adults.

We performed a detailed electrophysiology and fitting in these patients before and after the reimplantation in order to optimize their coding strategy. Nevertheless, their speech understanding with the current processor is way lower than in patients who got implanted in the last years. To explain this result, based only on few patients is more or less speculating. But on the basis of knowing the better maturation is dependent from early implantation we hypothesize that the less intelligent stimulation strategies in historic implants lead to a more “rough” auditory maturation. This potential “rough” auditory maturation is not able to raise from more soffisticated stimulation the same benefit for speech understanding as auditory system after maturation with more intelligent strategies.

Concluding from this hypothesis we assume that these patients are reduced to their artificial specific electric stimulation “code”, which lead to their individual speech and language development, being dependent from their “code”.

Any change in the represented frequency range can lead to a deprivation of the auditory perception and subsequent reduced auditory performance. This can be seen in patient 1 with a high difference in electrode position. Patient 2 on the contrary showed some improvement of the auditory performance with a similar electrode position. Patients with the new implant experience a stimulation of higher frequencies but lack the lower frequencies in comparison to the hearing with the first device. Therefore different parts of the cochlea are covered with active contacts. This leads to a worse speech understanding, which can be only partially compensated by other technological advances.

The hypothesis is that the different placement of the electrode stimulates areas along the auditory pathway up to the auditory cortex which are not activated so far and not related to hearing in this patient. This electrode mismatch leads to a decrement in performance which cannot be compensated by adaptive plasticity processes due to the fixed neuronal network in the auditory system of these patients. This is in contrast to postlingually deaf patients whose auditory system could develop with full frequency range auditory stimulation and shows no limited neuronal gate at the auditory pathway into the central auditory system. Their system therefore has kept the ability to adapt to changes in the artificial electrical stimulation. They can make use of the technological advancements of new implants used for reimplantation.

The hypothesis from this described experience in five patients is that for these patients rather long electrodes should be used to reach a deep insertion with a stimulation of the apical cochlea as they are used to have but also stimulate high frequencies within the basal cochlea although this is new to them.

Studies with a large cohort of patients are needed to further analyse the impact of other individual factors on auditory performance.
